# Lesions to Lateral Prefrontal Cortex Impair Lexical Interference Control in Word Production

**DOI:** 10.3389/fnhum.2015.00721

**Published:** 2016-01-20

**Authors:** Vitória Piai, Stéphanie K. Riès, Diane Swick

**Affiliations:** ^1^Department of Psychology, Helen Wills Neuroscience Institute, University of CaliforniaBerkeley, Berkeley, CA, USA; ^2^VA Northern California Health Care SystemMartinez, CA, USA; ^3^Department of Neurology, University of CaliforniaDavis, Davis, CA, USA

**Keywords:** Bayesian estimation, Broca’s area, competition, cognitive control, lexical selection, selective inhibition

## Abstract

Speaking is an action that requires control, for example, to prevent interference from distracting or competing information present in the speaker’s environment. Control over task performance is thought to depend on the lateral prefrontal cortex (PFC). However, the neuroimaging literature does not show a consistent relation between left PFC and interference control in word production. Here, we examined the role of left PFC in interference control in word production by testing six patients with lesions to left PFC (centered around the ventrolateral PFC) on a control-demanding task. Patients and age-matched controls named pictures presented along with distractor words, inducing within-trial interference effects. We varied the degree of competing information from distractors to increase the need for interference control. Distractors were semantically related, phonologically related, unrelated to the picture name, or neutral (XXX). Both groups showed lexical interference (slower responses with unrelated than neutral distractors), reflecting naming difficulty in the presence of competing linguistic information. Relative to controls, all six left PFC patients had larger lexical interference effects. By contrast, patients did not show a consistent semantic interference effect (reflecting difficulty in selecting amongst semantic competitors) whereas the controls did. This suggests different control mechanisms may be engaged in semantic compared to lexical interference resolution in this paradigm. Finally, phonological facilitation (faster responses with phonological than unrelated distractors) was larger in patients than in controls. These findings suggest that the lateral PFC is a *necessary* structure in providing control over lexical interference in word production, possibly through an early attentional blocking mechanism. By contrast, the left PFC does not seem critical in semantic interference resolution in the picture-word interference paradigm.

## Introduction

Speaking is a seemingly effortless task executed on a regular basis. Yet, speaking is a controlled action (e.g., Roelofs and Piai, 2011). As a broad term, control refers to regulatory/monitoring processes that ensure that our actions are in agreement with our goals, especially in the face of distraction (e.g., Posner and Petersen, 1990). We need to exert control over language production for various reasons. For example, speakers need to prevent interference from concurrent visual or auditory information present in their environment. Moreover, when retrieving words from long-term memory, associated information that is not relevant to the task at hand is also retrieved. Control must also be exerted over these interfering memory representations so the appropriate word can be selected (e.g., Badre and Wagner, 2007). Whereas the neuroanatomical characterization of core language-production processes has advanced considerably (e.g., Indefrey, 2011; Price, 2012), only recently has research been dedicated to the control aspects of production (e.g., Alario et al., 2006; Piai et al., 2013; Geranmayeh et al., 2014; Riès et al., 2014, 2015). In the present study, we examined the role of left lateral prefrontal cortex (PFC) in control over language production by testing stroke survivors with damage to left PFC on a control-demanding task. We varied the type of interfering information in order to increase the difficulty of picture naming, as we explain next.

A fruitful paradigm to investigate control functions implemented during speaking is picture-word interference (Rosinski et al., 1975). In this task, speakers are instructed to name pictures as fast and accurately as possible, while ignoring a distractor word superimposed on the picture. The distractor word can be manipulated to provide information (partially) congruent with the picture name (e.g., the picture of a carrot with the distractor “castle”, which overlap in their initial phonemes) or incongruent (e.g., pictured carrot, distractor “stamp”). Control functions are assumed to aid the production of the picture name in the presence of incongruent, competing information from the distractor word. In particular, naming pictures in the presence of distractor words likely requires *interference control*, an executive function involved in “suppressing a stimulus that pulls for a competing response so as to carry out a primary response, […] suppressing distractors that might slow the primary response […]” (Nigg, 2000, p. 222; for further discussion, see also Friedman and Miyake, 2004).

The type of relationship that the picture name bears with the distractor is an important determinant of people’s performance. For example, naming a picture with an unrelated distractor word (e.g., the picture of a carrot with the distractor “stamp”) is slower than naming a picture with a series of Xs superimposed (e.g., Glaser and Düngelhoff, 1984). In the neutral condition (XXX), only the picture name is activated by the picture stimulus, whereas with unrelated distractor words, both distractor word and picture name activate their representations, which then compete during word planning. We refer to this effect as the lexical interference effect. Furthermore, if the picture and distractor are from the same semantic category (e.g., pictured carrot, distractor: “radish”), picture naming is slowed down relative to an unrelated distractor. This effect is known as the semantic interference effect (e.g., Glaser and Düngelhoff, 1984). In this case, the semantic relation between picture and distractor makes the distractor a stronger competitor for the picture name relative to an unrelated word (e.g., Roelofs, 1992, 2003). By contrast, if the picture and the distractor share a phonological relationship (e.g., pictured carrot, distractor: “castle”), picture naming is faster relative to an unrelated distractor. This effect is known as the phonological facilitation effect (e.g., Schriefers et al., 1990). The phonological overlapping information between the distractor and the picture name pre-activates the picture name, facilitating its production.

Importantly, lexical and semantic interference possibly originate at different processing stages in language production. In particular, the semantic interference effect has been attributed to selection difficulty arising at the lexical level (e.g., Roelofs, 1992). Differently, the lexical interference effect could potentially reflect the operation of an early attentional blocking mechanism, which is more challenged in the presence of distractor words compared to the neutral condition (XXX; e.g., Roelofs, 2003; Roelofs et al., 2011). Whether the same brain region underlies the control mechanisms engaged in resolving these interference effects is unclear.

### Neuroanatomical Findings

The lateral PFC is known to be involved in broad aspects of top-down control over task performance (e.g., Petrides, 2005), such as monitoring and manipulating information, and regulating selection amongst competing representations (for reviews and meta-analyses, see e.g., Alvarez and Emory, 2006; Szczepanski and Knight, 2014; Yuan and Raz, 2014). With respect to picture-word interference, a number of neuroimaging studies have used different types of distractor words. However, no studies have examined lexical interference, and the evidence for differential activation in the PFC for semantic interference is mixed. Using auditory distractor words, one study found that the semantic interference effect was associated with modulations in the ventrolateral PFC activation (de Zubicaray and McMahon, 2009). In the other four studies, however, differential activation to distractors was observed in brain areas other than the lateral PFC, including the anterior cingulate and different structures within the left temporal cortex. For example, two studies found no increases in brain activity for the semantically related relative to the unrelated condition, only activity decreases in left temporal cortex (de Zubicaray et al., 2013; Piai et al., 2013). One of these studies also found activity increases in the anterior cingulate cortex when comparing semantically related and identity distractors (i.e., Piai et al., 2013). Finally, one study compared semantically related distractors to a neutral condition (XXX), which is an unusual comparison, and found anterior cingulate and superior frontal gyrus involvement (de Zubicaray et al., 2001). The involvement of lateral PFC in resolving the lexical interference effect therefore remains an open question.

Functional imaging studies can inform us about the engagement of brain areas in a particular function. However, claims regarding the necessity of those areas for a particular function are mainly possible by means of lesion-symptom investigations. Previous neuropsychological studies have primarily focused on the semantic interference and phonological facilitation effects. For example, a picture-word interference study conducted on aphasic individuals found increased semantic interference and increased phonological facilitation effects in aphasics relative to age-matched controls (Hashimoto and Thompson, 2010). However, lesion location for these patients was not provided. Biegler et al. (2008) also observed an increased semantic interference effect in two of their patients with left frontal lesions, whereas the third patient, with unknown lesion location, showed no semantic interference effect. To the best of our knowledge, there is no published picture-word interference study on a group of patients with well-characterized, focal PFC lesions. Furthermore, no neuropsychological study has examined the lexical interference effect (for lesion studies on other picture-naming tasks, see Schnur et al., 2006; Riès et al., 2014, 2015).

This brief review of the neuroimaging literature does not show a consistent relation between left PFC and the resolution of distractor interference in picture naming. Moreover, the current neuropsychological literature does not provide enough anatomical specification associated with impairments in interference control in picture naming. Finally, since the studies discussed above assessed the effect of competing *semantic* alternatives only, it remains unclear whether the left PFC is necessary in providing control over *lexical* interference in word production. Specifically, does the left PFC support control over word production processes in the presence of any distracting or competing word? Control over word production amidst competing (unrelated) alternatives would be indicated by increased interference from distractor words, which provide competing linguistic information, relative to a neutral condition (XXX), in which no competing linguistic information is present. If the left PFC is necessary for lexical interference control in word production, then the lexical interference effect should be larger in PFC patients relative to controls.

### The Present Study

We employed the picture-word interference task to examine the role of left PFC in interference control over word production. We tested six chronic stroke patients with damage to the left PFC and 13 age-matched controls on their picture-naming performance while varying the difficulty of word production by having semantically related, phonologically related, unrelated, and neutral (XXX) distractors. We assessed (1) whether patients and controls differed in overall performance; (2) whether distractor effects were present in the overall data and most importantly; and (3) whether the magnitude of each of these effects was comparable between patients and controls. Since we were interested in interference effects, we opted for maximizing the interference of distractors by presenting the picture and the distractor simultaneously (as is classically done in the picture-word interference task; e.g., Damian and Martin, 1999). Therefore, no manipulation of stimulus onset asynchrony was introduced.

The response times (RTs) were analyzed within a robust Bayesian estimation framework. We chose this type of analysis over more traditional null hypothesis significance testing for a number of reasons. Firstly, with this approach, we can provide richer information (i.e., an explicit posterior distribution) on how groups differ in the conditions under analysis and a more reliable estimate of this difference. Secondly, with an analysis of variance (ANOVA) approach, a multiple comparisons problem may exist (cf. Cramer et al., 2015). With the Bayesian approach adopted here, all parameters are estimated simultaneously, creating one fixed posterior distribution from which all effects are investigated, obviating the multiple comparisons problem. Finally, using Bayesian statistics, one can directly test whether there are no statistical differences between conditions. In null hypothesis significance testing, one could only reject the null hypothesis of no differences between patients and controls. However, one could not accept the null hypothesis that patients and controls do not differ in their behavior. Within a Bayesian framework, one can potentially also accept the null value (i.e., no difference between conditions compared) when the certainty in the estimate is high.

## Materials and Methods

The study protocol was approved by the Institutional Review Board of the Northern California Health Care System, following the Declaration of Helsinki. Participants gave written informed consent and were paid for taking part in the study.

### Participants

**Figure 1:**
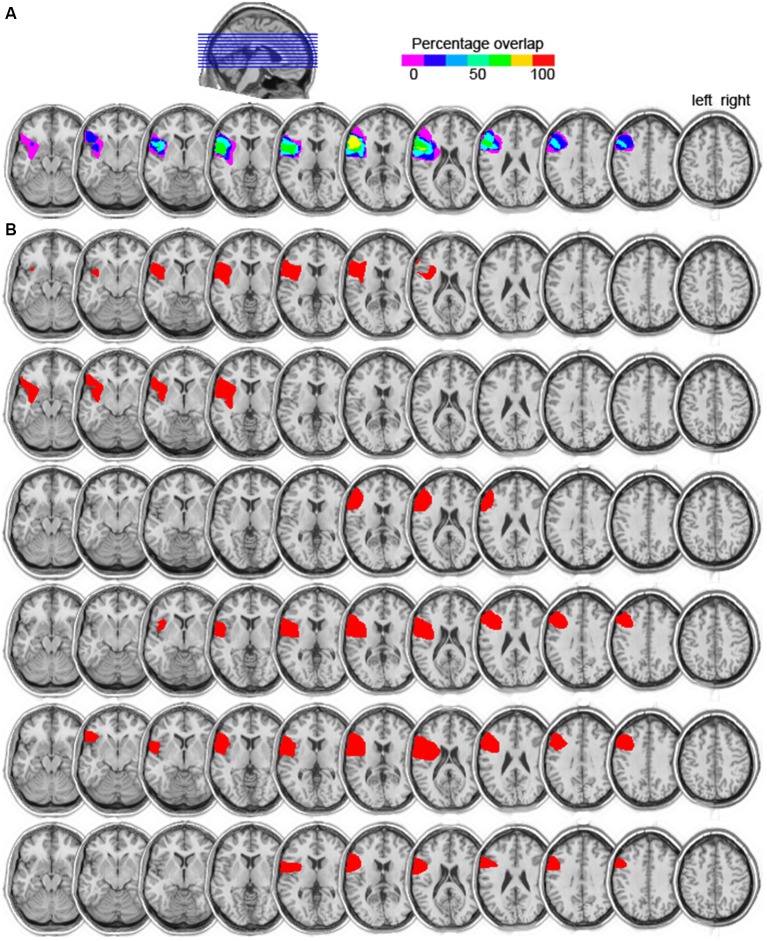
**(A)** Lesion overlap map of the six left prefrontal cortex (PFC) patients. The color scale indicates the amount of overlap in lesion locations, with magenta indicating that only one patient had a lesion in that particular region (i.e., 0% overlap). The maximum overlap, shown in yellow, indicates that five of the six patients had a lesion in that particular region (slices 6 and 7 from left to right). **(B)** Individual lesion reconstructions, shown in red. Each patient is shown in a subsequent row, from P1 (top) to P6 (bottom).

Six patients (five males, mean age = 66 years, mean education = 14) with focal damage to the left lateral frontal cortex participated. All were chronic stroke patients tested at least 1 year post stroke. Information on their handedness as well as on their language ability is shown in Table 1. All lesions were due to infarction in the precentral branch of the middle cerebral artery. The damage was centered on the pars triangularis of the ventrolateral PFC (five of the six patients), as shown in the lesion overlap map in Figure 1. Lesions were transcribed from CT or MRI scans onto corresponding axial templates by a neurologist for reconstruction. Patients’ performance on the Western Aphasia Battery (WAB; Kertesz, 1982), shown in Table 1 (for five of the six patients), indicated good language abilities in all five patients: An Aphasia Quotient (AQ) score of 93.8 or higher (out of 100) indicates performance within normal limits (Kertesz, 1982). Thus, our patients were not classified as aphasic. Additionally, 13 controls participated (five males; mean age = 66, *SD* = 7; mean years of education = 14, *SD* = 2.5). They were matched to the PFC patients for age (*t*_(12)_ < 1, *p* = 0.802), years of education (*t*_(8)_ < 1, *p* = 0.967), and handedness (one left handed participant in each group). None of the patients or control participants had a history of psychiatric disturbances, substance abuse, medical complications, multiple neurological events, or dementia.

**Table 1:** **Handedness, language testing, and lesion data of the patients**.

Patient	Handedness	Naming	Reading	AQ	MPO	Lesion vol (cc)
P1	Right	60	100	98.6	20	52
P2	Right	60	86	97.9	55	37
P3	Right	60	NA*	99	43	20
P4	Right	60	81	94.7	53	54
P5	Right	60	84	96.3	67	75
P6	Left	NA***	NA***	NA***	NA**	NA**

Note: Naming (maximum score = 60) and Reading (maximum score = 100) scores, as well as AQ (maximum score = 100), are measures from the Western Aphasia Battery (WAB). *P3 was not assessed on the WAB reading tests. However, the patient scored 100% in the reading assessment of sentences and paragraphs of the Boston Diagnostic Aphasia Examination. **Data not available. ***P6 was not assessed on language batteries as he was not aphasic. AQ, Aphasia Quotient; cc, cubic centimeter; MPO, months post stroke onset; vol, volume.

### Stimuli

Twenty-two pictures of objects were selected from Snodgrass and Vanderwart (1980). Each object was an exemplar of a different category. Three different types of distractor word were selected for each picture (materials were adapted from Taylor and Burke, 2002). In the related condition, distractors were taken from the same taxonomic category as the picture (e.g., picture: carrot, distractor: “radish”). In the phonological condition, a phonologically related (but semantically unrelated) word was chosen with the same two initial phonemes as the picture name (e.g., picture: carrot, distractor: “castle”). In the unrelated condition, the distractor was a semantically and phonologically unrelated word (e.g., picture: carrot, distractor: “stamp”). Finally, in the neutral condition, the picture was presented with XXX superimposed. Each distractor word appeared only once in the experiment. Distractors were matched for length (number of letters) and for frequency (*F*’s_(1,64)_ < 1)^1^. Pictures were presented once with each distractor, yielding a total of 88 experimental trials.

### Procedure

First, the participants were shown sheets of paper containing the pictures that were to appear in the practice and experimental trials, and were asked to name each one. If an incorrect response was given, the experimenter presented the correct name. Once this procedure was completed twice, testing on the computer began. Stimulus presentation and response recording were controlled by the Presentation Software (Neurobehavioral Systems, Albany, CA, USA). Each participant was seated in front of the monitor while wearing a headset with a microphone. The instructions informed participants that they would be naming pictures appearing on the screen and that, in addition, a word or XXX would be superimposed on each picture. They were instructed to ignore the distractor and name the picture on the screen as quickly as possible, avoiding errors or extraneous vocalizations. To begin each trial, the word “Ready” appeared on the monitor for 1500 ms. Next, a picture appeared simultaneously with a distractor stimulus and remained on the screen until a spoken response was made or 5000 ms had passed, and then the stimuli disappeared. The screen remained blank for 1000 ms before the following sentence appeared: “The correct answer is BLANK [the correct name of the object]. If you said BLANK, press the space bar.” This sentence appeared on the screen until the participant pressed the space bar or 3000 ms had passed. This self-evaluation procedure served unrelated purposes and these responses were not analyzed here. Note, however, that this procedure was presented for every participant and condition and as such, is not confounded with our distractor manipulation nor with participant group. The next trial followed 1000 ms later. There were seven practice trials followed by 88 experimental trials. RTs were measured by means of a voice key included in the Presentation software. The voice key was calibrated for each participant during the practice.

### Analysis

All data analysis was conducted using R (R Development Core Team, 2014) and “rjags” (Plummer, 2014). The experimenter monitored naming responses online. All trials with disfluent or incorrect responses were coded as errors and subsequently excluded from the RT analysis. Errors comprised between 0 and 3.8% of the patients’ responses and between 0 and 2.8% for the controls, with no significant differences between patients and controls for any of the distractor conditions (logistic regression, *p*’s > 0.09). Trials with voice-key triggering failure (5.3% of the total number of trials for patients and 3% for controls) or RTs shorter than 200 ms (one trial for one patient) were further excluded from the RT analysis. The RT data of both control and patient groups were not normally distributed. Given that the median is the best representative of central tendency with skewed data, participants’ median RTs were computed for each condition.

The RTs were analyzed with a hierarchical Bayesian estimation approach (Kruschke, 2015, available at http://www.indiana.edu/~kruschke/DoingBayesianDataAnalysis/Programs/SplitPlotJags.R). Under this approach, the data are modeled mathematically and belief is reallocated away from parameter values that are not consistent with the data in favor of parameter values consistent with the data. As such, inference from data does not rely on a *p* value. We used the 95% highest density interval (HDI) of the posterior distribution to decide whether to reject or accept the null value. The HDI summarizes a belief distribution such that values inside the HDI have a higher probability than values outside the HDI. If the HDI includes zero, then the difference between the estimated parameters describing the data in each condition is credibly zero. Thus, in this case, there is evidence in favor of the hypothesis of no differences between conditions.

The model we used can be related to an ANOVA in that we can examine the between-participant factor *group* (controls vs. patients), the within-participant factor *distractor effect* (lexical and semantic interference, and phonological facilitation) for each group, and their interaction (i.e., whether the magnitude of the distractor effects differ between patients and controls).

The data were modeled as coming from a *t* distribution. Each group by distractor combination is described with a *t* distribution with its own mean. The parameters describing the standard deviation (*SD*) and the tails of the distribution (i.e., the nu parameter) were the same for all group by distractor combinations so that their estimation is informed by data from both groups and all distractor conditions (e.g., Kruschke, 2013). The nu parameter represents the degrees of freedom of the *t* distribution, with small values indicating heavy tails and large values indicating a nearly normal distribution. The effect of each level of a factor on the data is described as a deflection away from the overall central tendency (baseline) of the data. These deflections respect the constraint that they must sum to zero. The variance of the deflections (i.e., the precision) is then estimated from the data (Kruschke, 2015). Whereas in the ANOVA framework, different comparisons rely on different error terms for computing the *F* ratio, in this Bayesian ANOVA, the parameters of all deflections are estimated simultaneously, creating one fixed posterior distribution. This posterior distribution is then used to examine the main, interaction, and simple effects of interest, obviating the multiple comparisons problem.

In this implementation, we have chosen prior distributions that are noncommittal and vague, having virtually no influence on the parameter estimates (Kruschke, 2013, 2015). The baseline value had a normal prior with a mean of 908 (the mean of the pooled data) and its precision was 1.0e−6 times the precision of the pooled data (i.e., 1.0e−11). The prior for the *SD* had a uniform distribution, with the lowest and highest values considered being the *SD* of the pooled data divided or multiplied by 1000, respectively. The prior on the nu parameter, describing the tails of the distribution, had a prior with an exponential distribution (mean = 29). This value is optimal for balancing between nearly normal and heavy-tailed distributions. This distribution assigns equal credibility to nearly normal and heavily tailed data (Kruschke, 2013). The priors for the effects (deflections from baseline) were also noncommittal, described with a normal distribution with a mean of zero. Its precision parameter had a gamma prior parameterized by the mean (5.0e−05) and 1.0e+6 times the *SD* (33) of the observed precisions of all group by distractor combinations, yielding shape = 2e−12 and rate = 4.6e−8. Importantly, choices of different priors and prior distributions did not affect the pattern of results obtained. The posterior distribution was approximated using a Markov chain Monte Carlo method with 100,000 samples. The chains were initialized based on the data, that is, the mean and *SD* of the pooled data, as well as of the factors group and distractor. In order to avoid problems with highly autocorrelated chains, the chains were thinned such that only each 50th step in the chain was used. The burn-in period was set to 2000 steps, that is, the first 2000 steps of the chain were discarded as they are not as well representative of the data as the later steps are. For completeness, we report the results of repeated measures ANOVA (uncorrected for multiple comparisons, cf. Cramer et al., 2015), with distractor type (related, unrelated, phonological, neutral) as a within-participants variable and group (patients, controls) as a between-participants variable.

## Results

The semantic interference and phonological facilitation effects were calculated relative to the unrelated condition (i.e., semantic interference: related—unrelated; phonological facilitation: phonological—unrelated), whereas the lexical interference effect was calculated as unrelated—XXX. In this way, interference is indicated by positive values and facilitation by negative values.

**Figure 2:**
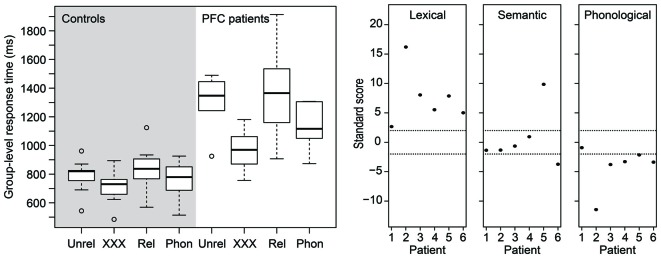
**(Left)** Box-and-whisker plot of the picture naming times of the age-matched controls (left panel) and of the patients (right panel). **(Right)** Standardized scores of the patients’ distractor effects. Dashed horizontal lines indicate 2 *SD*s (approximately the 95th percentile). PFC, prefrontal cortex; Phon, phonological; Rel, semantically related; Unrel, unrelated.

Figure 2 (left) shows a box-and-whisker plot of the participants’ median RTs. The right panel of Figure 2 shows how many *SD*s each patient’s performance is relative to the mean of the control participants for each effect of interest. Dashed lines indicate 2 *SD*s. It is clear from the figure that, for the lexical interference effect, all patients scored more than 2 *SD*s above the mean of the controls, suggesting relative impaired performance consistent across all patients. For the semantic interference effect, however, only two patients scored beyond 2 *SD*s, one above the mean and one below the mean of the control participants. Finally, for the phonological facilitation effect, five of the six patients scored more than 2 *SD*s below the mean of the control participants, suggesting that phonological distractors convey an additional benefit for the patients in picture naming relative to controls.

**Figure 3:**
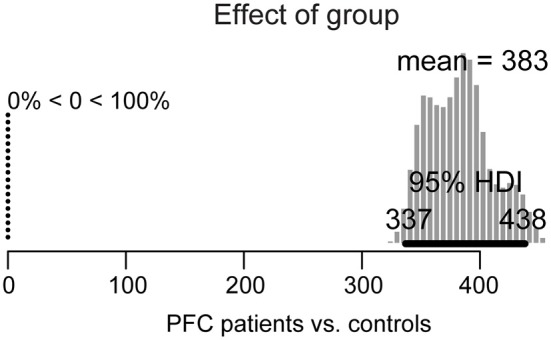
**Posterior distribution of differences in overall picture naming response times between lateral prefrontal cortex (PFC) patients and age-matched controls**. The 95% highest density interval (HDI) is marked by the horizontal bar on the bottom of the distribution. The mean of the parameter values for each distribution is indicated on the top, with the percentage of parameter values that are ±0 to the left.

**Figure 4:**
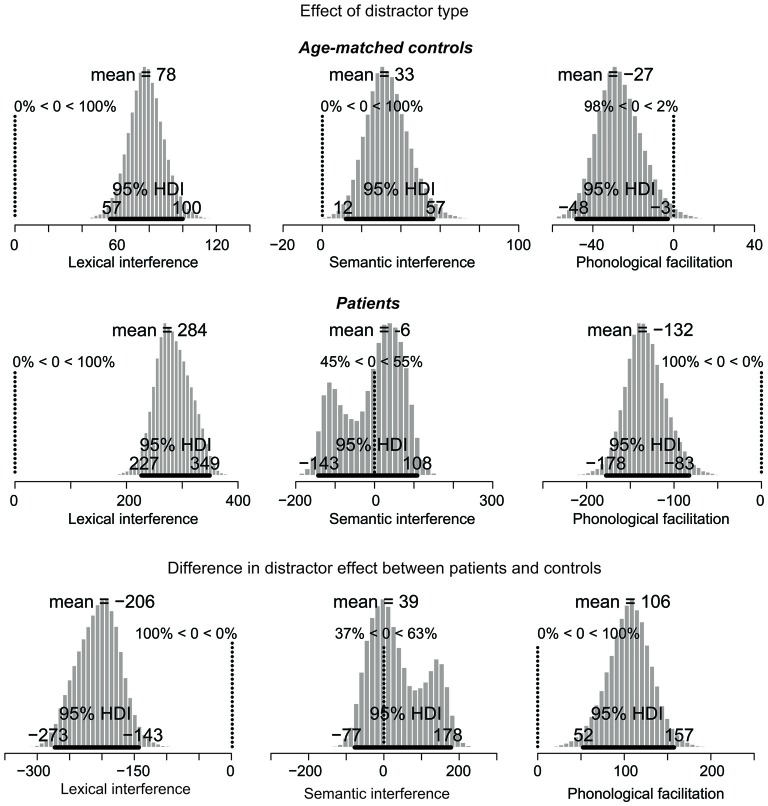
**Posterior distribution of differences in picture naming RTs as a function of distractor type for age-matched controls (top), lateral prefrontal cortex patients (middle), and for the difference between the two groups**. The 95% highest density interval (HDI) is marked by the horizontal bar on the bottom of the distribution. The mean of the parameter values for each distribution is indicated on the top, followed by the percentage of parameter values that are greater or smaller than zero. For the difference in effects (bottom), decreased performance (i.e., larger interference and smaller facilitation effects) in patients relative to controls are represented by values smaller than zero. Increased performance (i.e., smaller interference and larger facilitation effects) are represented by values greater than zero. Note: Scales differ across the graphs.

The results of the Bayesian estimation are summarized in Figures 3, 4. For each effect, a histogram is shown of 100,000 credible parameter values from the posterior distribution, given the data. The mean of the parameter values for each distribution is indicated on top. The 95% HDI is indicated for each histogram as a thick horizontal line below the distribution.

For the group effect (patients vs. controls), shown in Figure 3, the 95% HDI is clearly greater than zero (range: 337–438) and in fact, 100% of the credible parameter values of the difference in their performance is greater than zero. Thus, patients’ overall picture naming performance is credibly slower than in controls.

The next comparisons of interest are the distractor effects, shown in Figure 4. Interference effects are represented by positive values whereas facilitation effects by negative values.

The distractor effects in the control group are shown in the top row of Figure 4. For the lexical interference effect (unrelated vs. XXX, left panel), the 95% HDI clearly does not include zero (range: 57–100), with 100% of the credible parameter values greater than zero. Similarly, for the semantic interference effect (related vs. unrelated, middle panel), the 95% HDI is greater than zero (range: 12–57), with 100% of the credible parameter values greater than zero. Finally, for the phonological facilitation effect (phonological vs. unrelated, right panel), the 95% HDI does not include zero (range: −48 to −3), with 98% of the credible values smaller than zero. Thus, the lexical interference, semantic interference, and phonological facilitation effects are all credibly present in the data.

The distractor effects of the PFC patients are shown in the middle row of Figure 4. For the lexical interference effect (left panel), the 95% HDI clearly does not include zero (range: 227–349), with 100% of the credible parameter values greater than zero. By contrast, the semantic interference effect (middle panel) is not credibly present in the data: the 95% HDI includes zero (range: −143 to 108) with the credible parameter values almost symmetrically distributed below and above zero. Finally, for the phonological facilitation effect (right panel), the 95% HDI clearly does not include zero (range: −178 to −83), with 100% of the credible values smaller than zero. In summary, whereas the age-matched controls credibly showed all three classical distractor effects (lexical and semantic interference, and phonological facilitation), the PFC patients only showed credible lexical interference and phonological facilitation.

The comparisons of effect magnitude between patients and controls are shown in the bottom row of Figure 4. Decreased performance (i.e., larger interference and smaller facilitation effects) in patients relative to controls are represented by values smaller than zero. Increased performance (i.e., smaller interference and larger facilitation effects) are represented by values greater than zero. The lexical interference effect (left panel) is credibly larger in the patient group than in controls, with a 95% HDI between −273 and −143. In fact, 100% of the credible parameter values are smaller than zero. By contrast, the semantic interference effect is not credibly larger in patients than in controls, with the 95% HDI including zero (range: −77 to 178). Finally, patients have a credibly larger phonological facilitation effect than controls, with the 95% HDI between 52 and 157, and 100% of the credible values greater than zero.

The results of the ANOVA are presented in Table 2. These results largely converge with the results of the Bayesian analysis reported above. The only difference is the phonological facilitation effect in the controls, which was not significant in the ANOVA, but credible in the Bayesian analysis. However, with the Bayesian estimation we provide not only information on whether an effect is credible or not, but also a range of values for these effects that are most credible given the data.

**Table 2:** **Results of the repeated measures analysis of variance**.

	Mean diff (ms)	*F*	*t*	*df*	*P*	95% CI
**Effect**
Main: patient vs. control	415	34.97		1, 17	<0.001
Main: distractor		25.90		3, 51	<0.001
Interaction		11.41		3, 51	<0.001
**Effect**
Control: lexical	80		8.72	12	<0.001	60, 100
Control: semantic	46		3.49	12	0.004	17, 74
Control: phonological	18		−1.77	12	0.102	−40, 4
Patient: lexical	332		5.21	5	0.003	168, 495
Patient: semantic	75		0.81	5	0.452	−161, 311
Patient: phonological	172		−3.07	5	0.028	−315, −28
**Distractor: patients vs. controls**
Lexical	251		3.91	5.2	0.010	88, 414
Semantic	29		0.31	5.2	0.766	−265, 206
Phonological	172		2.70	5.3	0.040	10, 297

*Note: diff, difference*.

### The Effect of General Slowing

In order to assess whether the larger effect sizes in the patients than in the controls could be accounted for by general slowing, we conducted the following analysis. For each participant, we determined the RT ratio for each effect by calculating the RT difference between two conditions (lexical effect: unrelated minus neutral, semantic effect: related minus unrelated, phonological effect: phonological minus unrelated) and then dividing the difference by the RTs in the unrelated condition, which is common to all three distractor effects.

**Figure 5:**
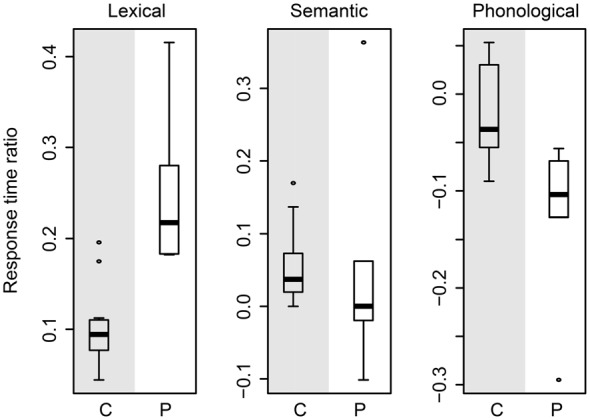
**Box-and-whisker plot of the response time ratios of the age-matched controls (gray-shaded panels) and of the patients (white-shaded panels) for the lexical (left plot), semantic (middle plot), and phonological (right plot) effects**. C, controls; P, patients.

Figure 5 shows a box-and-whisker plot of the RT ratios for age-matched controls (gray-shaded panels) and patients (white-shaded panels) for the three effects. The box-and-whisker plots show little overlap in the RT ratios for the lexical and phonological effects, indicating that these two effects cannot be explained by general slowing alone. That is, the lexical interference effect in patients is still larger than in the controls after correcting for general slowing. Similarly, the phonological facilitation effect in patients is still larger than in controls after accounting for the effect of general slowing. However, for the semantic interference effect, the RT ratios indicate similar magnitude in patients and controls. These results are compatible with a lack of a difference in semantic interference effect between patients and controls, as reported above.

## Discussion

This study is the first to examine picture-word interference in a group of patients with well-characterized left lateral PFC lesions, maximally overlapping in the ventrolateral PFC.

Patients were overall slower in picture naming than controls (for a similar finding in aphasic patients, see e.g., Biegler et al., 2008; Hashimoto and Thompson, 2010). Importantly, picture naming with a distractor word caused more interference for patients than for controls relative to a non-linguistic distractor (XXX). Furthermore, patients showed more phonological facilitation than controls. Finally, on a group level, patients failed to show a reliable semantic interference effect. These findings were evident from both the Bayesian estimation results and from the standardized scores of the effects for each patient. Below, we discuss each of these effects in more detail.

A novel finding from the present study was the increased lexical interference effect for patients relative to controls. In fact, all six patients were consistently impaired by distractor words relative to neutral, non-linguistic distractors. It has been argued that interference control is a significant cognitive construct, involved in multiple tasks and related to other cognitive abilities (Unsworth, 2010). However, its functional neuroanatomy in relation to word production has remained unclear. Previous neuropsychological studies using verbal tasks have often used the color-word Stroop task (e.g., Tsuchida and Fellows, 2013; Geddes et al., 2014), in which participants name the ink color of color names (e.g., *red* printed in red ink or *blue* printed in red ink). However, this task requires multiple repetitions of a few words (typically, 3–5 color names) and as such, is not ideal for investigating word production processes. Evidence that word planning depends on attentional control is now substantial (for review, see Roelofs and Piai, 2011) and recently, it has also been shown that interference control (also termed selective inhibition) plays an important role in picture-word interference (e.g., Shao et al., 2015). Our finding that competing linguistic information is especially problematic for all our patients with lateral PFC damage not only strengthens the previous relation between interference control and word production, but also further specifies that this type of control necessarily implicates the lateral PFC. This finding is in line with the neuropsychological literature suggesting a critical role of the left lateral PFC (and the ventrolateral PFC in particular) for interference control in verbal tasks (Tsuchida and Fellows, 2013; Geddes et al., 2014).

The semantic interference effect in the patients was highly variable. Descriptively, three patients had longer RTs for the related than the unrelated condition (i.e., semantic interference, patients 3, 4, and 5, with only one patient showing interference 2 *SD*s above the mean of the controls) and three patients showed the opposite effect (i.e., semantic facilitation, patients 1, 2, and 6, with one patient showing facilitation 2 *SD*s below the mean of the controls).

**Figure 6:**
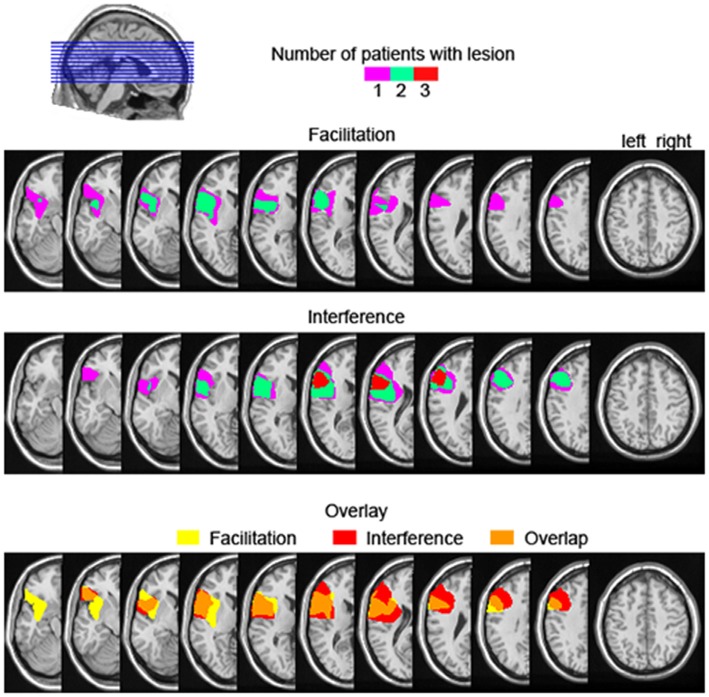
**Lesion overlap map of the three left prefrontal cortex patients showing descriptive semantic facilitation (upper) and semantic interference (middle), as well the lesion overlap between the semantic facilitation and interference groups (bottom)**. The color scale in the upper and middle panels indicates the number of patients with a lesion in a particular location. For the bottom overlay, lesion overlap of the facilitation group is given in yellow, for the interference group in red, and their overlap is shown in orange.

Facilitation from semantically related distractors is not a common finding in the literature. Wilshire et al. (2007) reported a case study of an anomic patient showing semantic facilitation with simultaneous presentation of picture and distractor (i.e., 0 ms stimulus onset asynchrony). However, given that all our six patients were not aphasic, comparisons with Wilshire et al.’s patient remain difficult. Other studies reporting semantic facilitation in picture-word interference had additional manipulations such as brief distractor pre-exposure, distractor masking, or low co-activation of distractors, which decreased the strength of the distractor as a competitor (e.g., Roelofs, 1992, 1993; Piai et al., 2012). It could be the case that, for the patients experiencing semantic facilitation, the dynamics of processing the distractor vs. the picture is altered for some reason, and the distractor is able to prime the picture name but not compete with it (for further discussion, see Piai et al., 2012). Additionally, it could be that the amount of ventrolateral PFC damage or the specific portion of the ventrolateral PFC damaged are critical in determining if and how much control processes will be impaired in the presence of semantic competitors. Rather than being a functionally uniform region, it has been argued that different portions of the ventrolateral PFC subserve different cognitive functions (Clos et al., 2013). Also, it could be the case that a larger lesion is more likely to encompass portions of the ventrolateral PFC that are critical to resolving competition among semantic alternatives. Variations in the extent of ventrolateral PFC affected could potentially explain the variable behavior of our patients regarding semantic effects. However, assessing these hypotheses directly is not possible in the present study. In Figure 6, we show the lesion overlap of the three patients showing facilitation vs. the three patients showing interference, as well as the overlap in lesion location between the two groups. As can be seen, although the three patients showing semantic interference have lesions maximally overlapping in the ventrolateral PFC, the group showing facilitation does not have a converging lesion maximum. Moreover, there seems to be great overlap in lesion location between the two groups. Thus, the extent to which left lateral PFC lesions affect the semantic interference effect in picture-word interference remains unclear at this point, but will hopefully be clarified in future investigations.

Using Bayesian estimation, we established that a zero difference in semantic interference in the patient group, on the *group* level, is a credible value given the data^2^. Thus, we can conclude that our patient group was not consistently impaired by semantic competitors. However, all six patients showed an increased lexical interference effect relative to controls. The dissociation between lexical and semantic interference in the patients’ behavior may indicate different types of control functions necessary for resolving lexical vs. semantic interference, which are differentially dependent on the lateral PFC. In the introduction, we related performance in picture-word interference to a control function enabling participants to suppress distractors that might slow down picture naming. However, the reason why distractors may hinder picture naming can be diverse. For example, in the picture-word interference literature, distractor effects exist that are caused by the distractor words themselves, independent of their semantic relation to the picture. The distractor-frequency effect is such an effect, in which pictures paired with low-frequency distractors are named more slowly than with high-frequency distractors (Miozzo and Caramazza, 2003). It has been shown that the semantic interference and distractor-frequency effects reflect different underlying operations (Scaltritti et al., 2015; for a computational model formalizing the parameters associated with these effects, see also Roelofs, 2003). The semantic interference effect is thought to emerge due to competition between the picture name and the distractor word during lexical selection (e.g., Roelofs, 1992; La Heij et al., 2006; Rahman and Aristei, 2010). By contrast, the distractor-frequency effect is explained by a reactive blocking mechanism that blocks the distractor word so that processing of the target picture is favored (Roelofs, 2003). The speed with which the distractor can be processed determines the speed of blocking (e.g., Roelofs et al., 2011). The blocking mechanism is an early attentional mechanism that operates independently from resolving semantic competition (e.g., Roelofs, 2003).

The lexical interference effect conceivably measures the engagement of this early attentional, blocking mechanism. The mechanism is always necessary in conditions with a distractor word, such as the unrelated condition, so that processing the picture is favored over distractor processing. In the neutral condition, no blocking is necessary because no distractor word is presented. So the lexical interference effect maximally captures the workings of the blocking mechanism. The semantic interference effect is not informative about the involvement of early attentional blocking demands, because the semantic and unrelated conditions both require blocking of the distractor word.

In our study, resolving lexical interference was impaired in all six left PFC patients whereas resolving semantic interference was not. This dissociation implies that the left PFC is critically involved in attentional distractor blocking so that picture processing is prioritized despite distracting information. But the left PFC is not necessarily required for resolving semantic competition between picture name and distractor word during response selection. This latter proposal is in line with recent evidence that the left lateral PFC is not always necessary to resolve interference from semantically related competitors (Riès et al., 2015). In this recent study comparing the performance of left PFC patients in two production tasks inducing semantic interference, Riès et al. (2015) found that left PFC patients had a larger semantic interference effect than controls in blocked-cyclic naming, a picture-naming task that requires proactive interference control due to blocked design and picture repetition. By contrast, in a continuous picture naming task in which pictures are not repeated (Howard et al., 2006), the same patients and controls had an interference effect of similar magnitude.

An additional finding of our study was that the phonological facilitation effect was larger in the patients than in the control participants, similar to the findings of Hashimoto and Thompson (2010) in their aphasic patients. It is well known that patients with word finding difficulties benefit from phonemic cues (e.g., Li and Williams, 1989). Although our patients performed within normal limits on the WAB and thus, were not aphasic as the patients in Hashimoto and Thompson (2010), it is common for this patient group (i.e., within normal limits-WAB) to still have subclinical language impairment beyond what would be expected for their age (e.g., Riès et al., 2015). In fact, such a suggestion finds support in our own data. As reported above, patients were overall slower than the controls. Perhaps even more importantly, in the XXX condition, in which the distracting information is neutral, patients were still slower (250 ms on average) than the controls. This finding is consistent with residual word-finding difficulties these patients often report. It is thus possible to explain the hyper phonological facilitation in these patients in that they might have developed a greater reliance on phonemic cues in comparison to controls when having word-finding problems. However, this account remains speculative at this point. Crucially, all six patients showed an increased lexical interference effect, providing evidence that any damage to the left lateral PFC will affect the ability to carry out picture naming in the presence of competing linguistic information.

## Conclusion

In conclusion, all six left PFC patients showed an increased lexical interference effect. The impaired performance of the patients in naming pictures in the presence of a competing distractor word (i.e., linguistic competing information) provides evidence that the left lateral PFC is a necessary structure in providing control over word production processes. This finding corroborates the evidence that word production requires attentional control and further specifies that interference control in word production necessarily implicates the lateral PFC.

## Author Contributions

Conceptualized and designed the experiment (DS); acquired the data (DS); analyzed the data (VP); wrote the article (VP, SKR, DS). All authors have approved the final version of the article and agree to be accountable for all aspects of this work.

## Conflict of Interest Statement

The authors declare that the research was conducted in the absence of any commercial or financial relationships that could be construed as a potential conflict of interest.

## Footnotes

^1^Unintentionally, one unrelated and two phonological distractors chosen were adjectives rather than nouns (“tactic”, “antique”, “frozen”), and one distractor was not a concrete noun (“dream”). We note that the pattern of results was the same whether we included or excluded these items, so the results reported comprise all distractors.

^2^We note that accepting the hypothesis of zero difference between conditions is only possible when there is high enough precision in the estimation of the parameters. In that case, one defines an interval of parameter values that are negligibly different from the null value for practical purposes (e.g., an effect of 10 ms may be deemed negligibly different from 0 ms). When all the credible parameter values fall within this interval, the null value is accepted. Unfortunately, our sample size is not large enough to yield very precise parameter estimations.
